# Inbreeding Alters Activities of the Stress-Related Enzymes Chitinases and β-1,3-Glucanases

**DOI:** 10.1371/journal.pone.0042326

**Published:** 2012-08-03

**Authors:** Roosa Leimu, Lena Kloss, Markus Fischer

**Affiliations:** 1 Department of Plant Sciences, University of Oxford, Oxford, United Kingdom; 2 Institute for Biochemistry and Biology, University of Potsdam, Potsdam, Germany; 3 Department of Community Ecology, Helmholtz Centre for Environmental Research - UFZ, Halle, Germany; 4 Institute of Plant Sciences, University of Bern, Bern, Switzerland; Centro de Investigación y de Estudios Avanzados, Mexico

## Abstract

Pathogenesis-related proteins, chitinases (CHT) and β-1,3-glucanases (GLU), are stress proteins up-regulated as response to extrinsic environmental stress in plants. It is unknown whether these PR proteins are also influenced by inbreeding, which has been suggested to constitute intrinsic genetic stress, and which is also known to affect the ability of plants to cope with environmental stress. We investigated activities of CHT and GLU in response to inbreeding in plants from 13 Ragged Robin (*Lychnis flos-cuculi*) populations. We also studied whether activities of these enzymes were associated with levels of herbivore damage and pathogen infection in the populations from which the plants originated. We found an increase in pathogenesis-related protein activity in inbred plants from five out of the 13 investigated populations, which suggests that these proteins may play a role in how plants respond to intrinsic genetic stress brought about by inbreeding in some populations depending on the allele frequencies of loci affecting the expression of CHT and the past levels of inbreeding. More importantly, we found that CHT activities were higher in plants from populations with higher levels of herbivore or pathogen damage, but inbreeding reduced CHT activity in these populations disrupting the increased activities of this resistance-related enzyme in populations where high resistance is beneficial. These results provide novel information on the effects of plant inbreeding on plant–enemy interactions on a biochemical level.

## Introduction

The rapid changes in environmental conditions due to human activities expose plants to an elevated environmental stress including drought, salinity, extreme temperatures, and altered interactions with herbivores and pathogens. In addition to environmental stress plants can suffer from intrinsic genetic stress, which has been suggested to arise as inbreeding changes the genetic architecture of plants and their populations [1]. This genetic stress is becoming increasingly important especially in natural populations where inbreeding is ubiquitous because populations are commonly getting smaller due to human activities [2]. In addition to resulting in reduced fitness, i.e., inbreeding depression, inbreeding can also affect the ability of plants to cope with environmental stress. Inbreeding and environmental stress have been shown to reduce plant fitness more in combination than alone [3], [4]. Furthermore, plant inbreeding can alter interactions with herbivores and pathogens. Recently, a number of studies have reported effects of plant inbreeding on herbivore resistance and tolerance, plant quality for herbivores, herbivore performance, and pathogen infection rates at phenotypic level [5]–[13]. These effects varied from positive to negative, i.e., from inbreeding benefits to inbreeding depression.

Despite increasing interest in the effects of plant inbreeding on plant-enemy interactions, the impact on biochemical mechanisms and processes underlying plant resistance to pathogens or herbivores remains largely unexplored. One of the first attempts has been trying to understand how inbreeding influences volatiles that mediate plant-insect interactions [14]–[16]. A recent study by Kariyat et al [16] demonstrated that inbreeding results in suboptimal volatile emission patterns increasing apparency to herbivores and reducing the attraction of natural enemies of herbivores. Inbreeding has also been shown to affect levels of herbivory due to changes in nutrient levels [11]. Yet, direct effects of inbreeding on plant defence compounds or pathways have not been investigated.

One way in which plants respond to extrinsic environmental stress is by elevated activity of pathogenesis-related (PR) proteins, chitinases (CHT) and β-1,3-glucanases (GLU) [17]–[22]. These proteins are generally referred to as stress-proteins and they are known to play an essential role in plant defence against natural enemies [17]–[23]. The expression levels of PR proteins are known to increase in response to abiotic stress such as heavy metals, salt, and drought [24]. Whether inbreeding affects their activities is, however, not understood. Understanding how inbreeding affects PR protein activities is central for assessing the effects of inbreeding on plant resistance against natural enemies. Activities of PR proteins are often induced as response to pathogen or herbivore damage, but it has been shown that, for example, more resistant lines of tomato also show higher constitutive levels of CHT and GLU [25].

Here we investigated the role of GLU and CHT in plant resistance against pathogens and herbivores, and whether inbreeding compromises the defensive role of these PR proteins by comparing levels of GLU and CHT between inbred and outbred F2 Ragged Robin (*Lychnis flos-cuculi*) individuals from 13 natural populations in a greenhouse experiment. We further tested whether inbreeding affects on these PR proteins depend on past levels of herbivory or pathogen infection experienced in the field.

## Results

**Figure 1:**
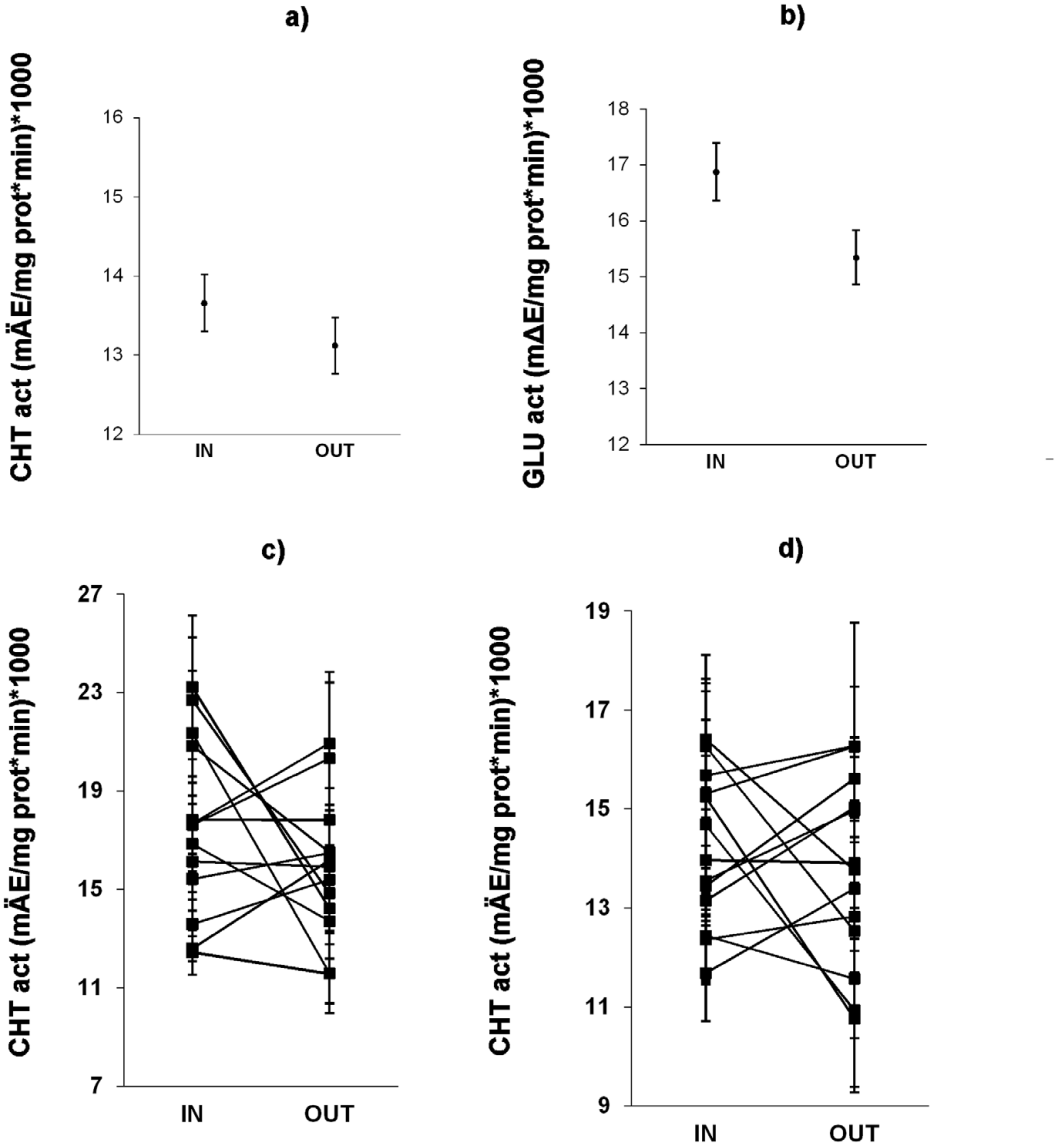
Inbreeding effects on PR protein activity in Ragged Robin. Effect of experimental inbreeding on constitutive activity of a) chitinases (CHT) and b) β-1,3-glucanases (GLU). Variation in c) CHT and d) GLU activities among plants from the 13 investigated populations. Enzyme activities are expressed as average values (± standard error) across the populations (1a, 1b) or per population and cross (1c, 1d) in increase of absorbance at 600 and 550 nm per mg protein and per min ×1000 (GLU and CHT, respectively).

Experimentally inbred plants had statistically significantly higher constitutive activity of CHT compared to outbred plants (Table 1; Fig. 1a). Average constitutive activity of GLU also tended to be higher in inbred compared to outbred plants, but this difference was not statistically significant (non-significant main effect) (Table 1; Fig. 1b). However, the effects of inbreeding on GLU activity varied among populations: inbred plants had higher GLU activity than outbred plants in some of the populations while in other populations the opposite was true as indicated by the significant population × cross interaction (Table 1). Inbreeding effects on CHT activity also tended to vary among populations (Table 1).

**Table 1:** The effects of experimental inbreeding (cross) and past levels of pathogen and herbivore damage on constitutive activities of β-1,3-glucanases (GLU) and chitinases (CHT) in plants from 13 Ragged Robin populations.

	Source of variation			
**GLU**	*Fixed effects*	*d.f.*	*F*	*P*
	Cross	1, 11.5	1.38	0.263
	Herbivore damage	1, 10.8	0.01	0.939
	Pathogen infection	1, 10.9	3.13	0.105
	*Random effects*	*χ^2^*	*P*	
	Population	1.70	0.096	
	Population × Cross	6.8	0.004	
	Family (population)	0.2	0.327	
**CHT**	*Fixed effects*	*d.f.*	*F*	*P*
	Cross	1, 10.8	7.29	0.021
	Herbivore damage	1, 10.6	5.55	0.039
	Pathogen infection	1, 11	4.26	0.063
	Cross × Herbivore damage	1, 11.4	6.39	0.0274
	*Random effects*	*χ^2^*	*P*	
	Population	0.30	0.293	
	Population × Cross	1.90	0.084	
	Family (population cross)	0.80	0.185	

**Figure 2:**
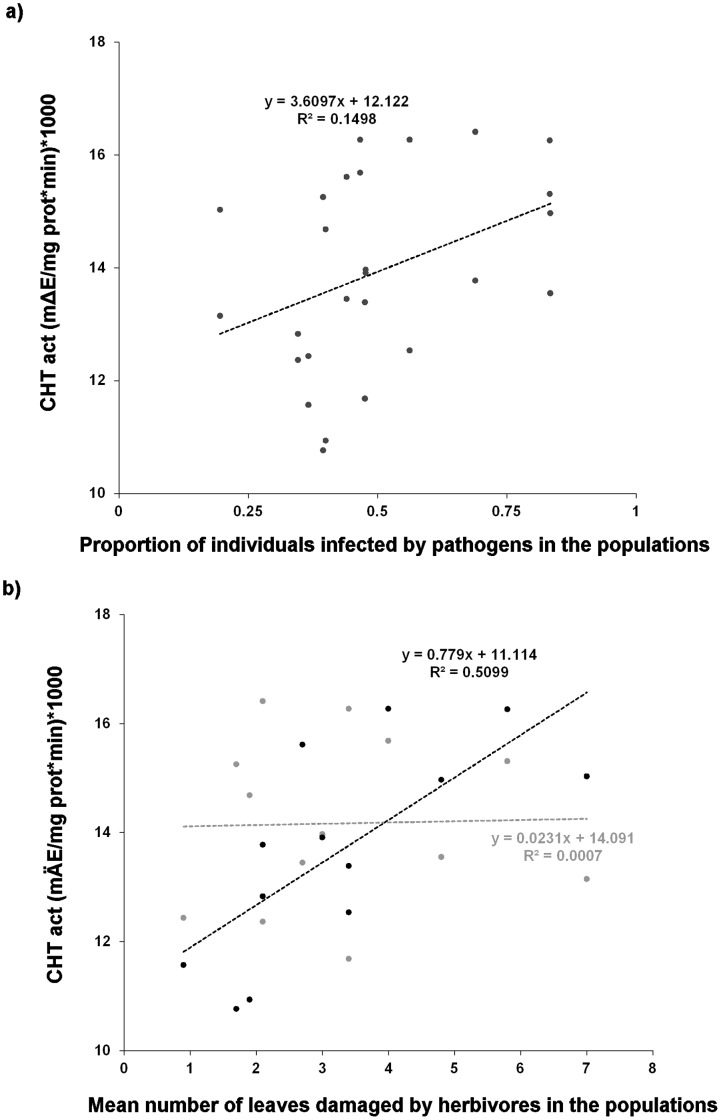
Effects of past herbivory and pathogen infection levels on PR protein activity in Ragged Robin. Constitutive CHT activities for plants from the 13 Ragged Robin populations a) in relation to levels of pathogen infection in the populations and b) inbreeding effects on CHT in relation to levels of herbivory in the populations. The gray dots denote population means for experimentally inbred plants and black dots denote population means for experimentally outbred plants (2b).

CHT activities were higher in plants from populations that had experienced higher levels of herbivory and pathogen infection in the past (Table 1; Fig. 2). The impact of past herbivory on CHT activity differed, however, between inbred and outbred plants as indicated by the significant cross × herbivory interaction (Table 1; Fig. 2b). CHT activity increased with increasing level of herbivory experienced in the past, but only for the outbred plants. CHT activity in inbred plants was not associated with past level of herbivory (Table 1; Fig. 2b).

## Discussion

Inbred Ragged Robin individuals had, on average, higher constitutive PR protein activity than outbred individuals, suggesting higher resistance. This does not support the idea that inbreeding results in reduced resistance. Increased PR protein activity due to inbreeding may, in fact, result in indirect beneficial effects in terms of higher resistance depending on other biochemical effects of inbreeding. Alternatively, the consequent constitutive over-expression of CHT in inbred plants may affect their ability to induce this protein as response to herbivore and pathogen attack, or environmental stress, therefore reducing their resistance or stress tolerance. In line with this idea, inbreeding has been shown to elevate constitutive volatile emission while reducing herbivore-induced emission thereby resulting in an overall suboptimal emission pattern [16]. The exact mechanism underlying the over-expression of CHT in inbred plants, and its consequences for plant resistance and fitness, is not known. One explanation for this over-expression can be increased homozygosity of deleterious alleles at loci affecting the expression of CHT in inbred plants.

In general, inbreeding depression is known to vary among genotypes within population, among populations, and to depend on the environmental conditions [3], [4], [26]. The few studies that have investigated inbreeding effects on plant-enemy interactions in multiple populations have found substantial among-population variation in the effects of inbreeding on, for example, damage levels [10]. It is important to note that, despite the overall increase in PR protein activity due to inbreeding, when investigating variation among populations we found an increase due to inbreeding only in plants from some of the 13 investigated Ragged Robin populations while in plants from other populations inbreeding had no or negative effects on PR protein activity. These among-population differences in response to inbreeding are likely to be due to variation in past levels of inbreeding and in the frequencies of alleles associated with PR protein activity due to genetic drift. In line with this idea, we have previously demonstrated that inbreeding effects on resistance (measured as inverse of damage) and fitness vary among populations depending on their genetic history [10]. We hypothesized that, if CHT and GLU are related to herbivore and pathogen resistance in Ragged Robin, their constitutive activities should be higher for populations with higher levels of damage by natural enemies, provided selection for high resistance is not constrained by excessive metabolic costs. In line with these predictions, we found higher overall constitutive activity of CHT in plants from populations with higher herbivory or pathogen infection levels. Constitutive levels of CHT and GLU have been demonstrated to be higher in plants that are more resistant against natural enemies [25]. Our results, thus, suggest that natural selection has favoured increased constitutive activities of CHT in populations with higher herbivory or pathogen infection levels. We have previously shown that experimentally inbred individuals of Ragged Robin suffer higher-than-average levels of damage in populations subject to higher past levels of herbivory [10]. This implies reduced resistance due to inbreeding in populations where selection should favour high resistance. In populations with lower levels of herbivory, inbreeding had a direct and negative impact on plant fitness rather than resistance [10]. The results of our analysis of CHT activity confirm these findings at a biochemical level: outbred plants from populations with high past levels of herbivory had higher CHT activity than inbred plants whilst the opposite was true for plants from populations with low past levels of herbivory. Therefore, inbreeding appears to disrupt increased activity of this resistance-related enzyme in populations with high levels of damage by natural enemies, i.e., where selection should favour high resistance.

Recently, there has been a considerable and increasing interest in investigating inbreeding effects at different ‘omic’ levels [1]. Understanding how inbreeding affects transcriptomics, proteomics or metabolomics can help us to unravel the architecture and causes of inbreeding depression [1]. The first ‘omic’ studies of inbreeding have demonstrated that inbreeding can affect gene expression and protein function thereby influencing genetic and physiological processes [1], [27]–[29]. It has been proposed that molecular and biochemical responses to inbreeding result from general disturbance of cellular homeostasis [1], [27]–[29]. Inbreeding has also been shown to influence the same cellular processes as aging and environmental stress [1]. For example, recent studies on *Drosophila melanogaster* demonstrated that inbreeding induced heat shock proteins (HSP), a mechanism by which cells cope with stressful conditions [30], [31]. This suggests that these proteins are involved in coping with inbreeding in a similar manner as with extrinsic environmental stress [30], [31]. These data suggests that the expression of the genetic load due to inbreeding induces molecular responses that can counteract the deleterious effects of inbreeding [1]. So far these ideas have not been investigated in plants. Thus, it is yet unknown whether stress proteins, such as CHT and GLU, generally respond to inbreeding in order to allow plants to better cope with the stress brought about by inbreeding. The fact that the activities of GLU and CHT were, on average, higher in experimentally inbred F2 Ragged Robin individuals when compared with outbred individuals, although not significantly so for GLU, suggests that inbreeding may have a similar effect on the expression of the two PR proteins as extrinsic biotic or abiotic stress. This provides the first evidence for inbreeding effects on PR proteins and suggests that these stress proteins may not only function as response to environmental stress but also to genetic stress. More studies are, however, needed to confirm the role of PR proteins as response to inbreeding, and to unravel the mechanisms by which inbreeding affects PR protein activity.

### Conclusions

Our results demonstrate that inbreeding can alter the expression of stress proteins, chitinases (CHT) and β-1,3-glucanases (GLU). Inbreeding resulted in suboptimal constitutive expression of these stress proteins in plants from populations with high herbivore and pathogen pressure. An elevated activity of CHT and GLU due to inbreeding also suggests that these proteins may be involved in coping with inbreeding. More studies are needed to confirm the role of PR proteins as response to genetic stress caused by inbreeding, and to unravel the mechanisms by which inbreeding affects PR protein activity.

## Materials and Methods

We used plants from 13 Ragged Robin, *Lychnis flos-cuculi*, L. (Caryophyllaceae), populations located in calcareous fens in NE Switzerland. The population sizes range from ca 200 to 50000 individuals and the levels of inbreeding vary from F_IS_ 0.335 to 0.585 [32]. A number of generalist and some specialist herbivores, and the anther smut fungus, *Microbotryum violaceum* (Microbotryaceae), attack the plants in the field [33], [34]. Data on herbivore damage levels and pathogen infection rates three years prior to the experiment were obtained from previous studies [33], [35], [36]. *Lychnis flos-cuculi* is not threatened or protected in Switzerland and none of our study sites were on protected land, therefore, no specific permits were required for the described field studies. However, as all study sites were on privately owned land, permission to work on their land was obtained from all landowners and land users before the start of the project.

Initially, in each population twelve randomly selected plants were assigned as maternal plants and bagged in 2000. To obtain inbred F1 offspring altogether 156 maternal plants were selfed by hand pollinations between two flowers of the same plant. To obtain outbred F1 offspring the 156 plants were hand-pollinated with another randomly selected plant from the same population. For the outcrosses each plant served once as maternal plant and once as paternal one. Different pollen donors were used for different maternal plants. The distances between pairs of crossed plants ranged from 5 to 10 meters in the field. The pollinated flowers were bagged and mature capsules were collected when the seeds had ripened. The seeds were germinated and two randomly selected seedlings per fruit were transplanted and grown in the greenhouse until they flowered.

The F1 plants resulting from selfing were further selfed by hand-pollination to obtain the inbred F2, i.e., seeds resulting from two generations of selfing. The F1 plants resulting from outcrossing were further outcrossed with unrelated offspring of plants from the same population to obtain the outbred F2, i.e., seeds resulting from two generations of outcrossing within the same population. For our experiment, we used these seeds to grow inbred and outbred F2 plants, i.e., for each of the 13 populations of origin we used plants that resulted from two generations of inbreeding and plants that resulted from two generations of outcrossing. Using the F2 rather than the F1 plants has the advantage of reducing potential maternal environmental carry-over effects. We sowed seeds from 40 inbred and 54 outbred families in the greenhouse to obtain a total of 108 inbred and 107 outbred F2 plants (6 to 11 replicates per population) and grew the plants for four months, until when the plants were fully-grown and about to start flowering. All plants were well watered every day or every second day and grown under natural light conditions corresponding to the full-sun wet-grassland conditions in natural populations. We harvested 300±1 mg (Acculab, Edgewood, USA) of leaf material from the inner parts of all rosettes of a plant using flame sterilized scissors and froze it in liquid nitrogen immediately after weighing. The samples were stored at −80°C until biochemical analysis.

We analysed the activities of two resistance-related hydrolysing enzymes, chitinases (CHT) and β-1,3-glucanases (GLU). CHT catalyses the hydrolysis of chitin, which is the predominant constituent of fungal cell walls, of nematode eggs, and of the mid-gut layers of insects [22]. GLU, in turn, hydrolyzes callose and glucan polymers of pathogen cell walls and can synergistically enhance CHT activity, when chitin layers are buried by β-glucans [20], [22]. Both of these two enzymes have been demonstrated to be ubiquitous in responses to damage due to different causes [21], [37], [38].

Enzyme activities of the chitinases (CHT) and β-1,3-glucanases (GLU) were colorimetrically assayed according to the method of Wirth and Wolf [39] and Venisse *et al*. [40] with some modifications. We extracted the enzymes by grinding 300±1 mg plant material per plant in 3 ml of the extraction buffer described in Venisse *et al*. [40] with a mortar and pestle on ice. Homogenates were centrifuged at 16,000×*g* at 4°C for 20 min and supernatants were used immediately for enzymatic activity assays. We determined the activities of CHT and GLU twice for each sample with carboxymethyl-Chitin-Remazol Brilliant Violet (CM-Chitin-RBV) and carboxymethyl-Curdlan-Remazol Brilliant Blue (CM-Curdlan-RBB) as their respective substrates (Loewe Biochemica, Sauerlach, Germany).

For the enzymatic reaction, we prepared 100 µl of CM-Chitin-RBV (200 µg/ml) or CM-Curdlan-RBB (400 µg/ml) in 400 µl sodium acetate reaction buffer (200 mM, pH 5) pre-incubated at 37°C for 10 min and then added 200 µl of plant extract to the substrate solution. The mixture was incubated at 37°C for 30 min and the enzymatic digestion was stopped immediately by addition of 300 µl of 2 M HCl. For the reference, the reaction was stopped immediately with HCl after the addition of the plant extract. Non-digested substrate was precipitated after 10 min of incubation on ice and then separated from the digested substrate by centrifugation at 10,000×*g* for 10 min. CHT and GLU activities were calculated from the difference of absorbance (550 and 600 nm, respectively) between the incubated sample and reference. Data were expressed as the increase of absorbance per min and per mg protein ×1000. The protein content of the plant extracts diluted in extraction buffer (1∶10 v/v) was measured in duplicate using the dye solution Roti-Nanoquant (Carl Roth, Karlsruhe, Germany) following manufacturer instructions. Bovine serum albumin (0.1 mg/ml) served as standard for the calibration curve.

### Statistical Analyses

We conducted general linear mixed models to test for effects of experimental inbreeding on the activities of the two pathogenesis-related enzymes, CHT and GLU and how these effects vary among populations using the MIXED procedure of SAS statistical package version 9.1. Cross (IN or OUT) was treated as a fixed factor, and population, and family nested within population as random factors. Herbivory or pathogen infection the populations experienced in the past were included in the model as continuous variables to investigate whether they explain among-population variation in inbreeding effects on the enzyme activities. We used the Kenwardroger option to obtain approximate degrees of freedom for the denominators of each test statistic [41]. We used AIC-values to select the variance-covariance matrix structure and the models of best fit [41]. Significance of random factors was tested using the chi-square test (df = 1) of differences in the 2× log likelihood values of a given random factor included versus excluded from the model [41]. CHT was log-transformed to meet model assumptions.
